# To: The efficacy of high-flow nasal cannula *versus* non-invasive mechanical ventilation in preventing reintubation in patients at high risk of extubation failure: systematic review and meta-analysis with trial sequential analysis

**DOI:** 10.62675/2965-2774.20260223

**Published:** 2026-08-21

**Authors:** Yeşim Şerife Bayraktar

**Affiliations:** 1 Selçuk University Faculty of Medicine Division of Intensive Care Department of Anaesthesiology and Reanimation Konya Turkey Department of Anaesthesiology and Reanimation, Division of Intensive Care, Selçuk University Faculty of Medicine - Konya, Turkey.

**TO THE EDITOR,**

I read with interest the systematic review and meta-analysis by Molina et al. comparing high-flow nasal cannula (HFNC) with noninvasive ventilation (NIV) in patients at high risk of extubation failure.^(1)^ While the work is methodologically extensive, I wish to raise a fact regarding the effect measure used, as I believe it materially affects the interpretation of the results.

Throughout the forest plots (Figures 2, 4 and 5), the pooled estimates are expressed as the "risk ratio (non-event)" – that is, the ratio of the probability of *not* experiencing the outcome, rather than the conventional ratio of the event itself. Two consequences follow. First, the directional axis labels become internally inconsistent: in the subgroup ventilated for more than 7 days, for example, HFNC carried more reintubations than noninvasive ventilation (NIV) (5/20 *versus* 0/20), yet the corresponding estimate sits on the "favours HFNC" side of the axis. Second, and more important, this choice obscures a consistent mortality signal. According to the authors’ own GRADE table, intensive care unit death occurred in 42/844 patients (5.0%) receiving HFNC *versus* 63/853 (7.4%) receiving NIV, and hospital death in 100/844 (11.8%) *versus* 119/853 (14.0%). On the conventional event scale, the point estimate for intensive care unit mortality therefore favors HFNC (risk ratio of approximately 0.67). The reported non-event risk ratio of 1.03 (95%CI 1.00 - 1.07), described in the GRADE table as "2 more per 1,000," conveys the opposite impression despite fewer deaths in the HFNC arm. I would be grateful if the authors could re-present mortality and the remaining dichotomous outcomes on the standard event scale, so that both direction and magnitude can be appraised transparently.

A second, related point concerns heterogeneity. The authors acknowledge that excluding the single trial by Magdy et al. reduced I^2^ for reintubation from 65% to zero, identifying it as the dominant source of variability;^(2)^ the primary outcome therefore rests heavily on one chronic obstructive pulmonary disease trial. This fragility is compounded by clinical heterogeneity within the comparator arm itself: the "NIV" node pools mechanistically distinct modalities – helmet NIV, bilevel positive airway pressure, and continuous positive airway pressure, which provides no inspiratory support^(3)^ – across populations as varied as sepsis, hypercapnic chronic obstructive pulmonary disease, and obesity.^(4)^ Combined with the inconsistent definitions of "high risk" and the low prevalence of obesity (15.1% and 15.9%), these factors caution against a firm conclusion of equivalence, particularly for Western intensive care populations.

I commend the authors for addressing an area of genuine clinical uncertainty and hope these observations are received in the constructive spirit in which they are intended.
